# The Open-Air Treatment of Phthisis

**Published:** 1898-05-28

**Authors:** 


### THE OPEN-AIR TREATMENT OF PHTHISIS.

It does not speak well for the healthiness of our dwelling-houses that the mere placing of a patient in the open air should relieve him of much of his distress and remove many of his most important symptoms. Such, however, seems to be the case in regard to phthisis.

The open-air treatment of pulmonary tuberculosis is a matter which is forcing itself strongly upon professional attention at the present time, for its efficiency is well vouched for by many well-known physicians.

The last pronouncement in its favour comes from Dr. Theodore Williams, who has for a long time back advocated the treatment of phthisis by residence at high altitudes. He, however, in no way modifies his views as to the utility of high altitudes. There is in them, no doubt, something specific, connected probably with the development of compensatory changes in the healthy lung, connected possibly with those changes in the condition of the blood which are induced by mountain air. Nevertheless, the *régime* adopted by those undergoing the Alpine cure is to a large extent the same as in that by open air; and although it may be admitted that high altitudes are the more efficacious, it is clear that the life in the open air, which is the active agent in the "cures" at a lower elevation, also play no mean part in the striking results obtained at Davos, in the Andes, and elsewhere.

The position to which we have now reached seems to be this, that true as it is that in the glorious sunshine in the Alps the open air is more agreeable, and that in the keen dryness of those favoured health resorts the stimulating effects of the mountain air make it easier for a due amount of exercise to be taken, yet that, so far as living in the open is concerned, it is possible for an invalid to get fresh air during the winter months in England much as elsewhere if proper precautions be taken. In the choice of a place it is to be remembered that fog is the great difficulty. Frost, snow, rain, and hail are borne without material inconvenience; even dampness, except in certain catarrhal cases, does not do much harm. Fog is, however, a trouble, and the liability of towns to fog, together with other obvious reasons, will no doubt always render it desirable to carry out the open-air treatment of phthisis in the country. The leading principles, as worked out at Görbersdorf and Falkenstein, are that the consumptive should pass the greater part of the day in the open air, protected from weather, and as a rule in the prone position, and that at night he should sleep with windows open. At the institutions devoted to this treatment, it is carried out in covered terraces or in pavilions, some of which are made to rotate so as to afford protection from the wind. In the covered terraces, from which the drifting rain and snow are kept out by curtains, and the too intense sun by blinds, the patients lie out for from seven to eleven hours per day, only moving for meals and exercise and going indoors at night. For many reasons it is far better to lie down than to sit, especially in the cold weather. This life of reclining in the open air hardens the invalid against fresh cold, increases the appetite, promotes sleep, reduces night sweats, and, most important of all, lowers pyrexia. "I know of no more certain means of reducing the high temperature of acute tuberculisation, no matter what the condition of the lung, than the open-air treatment, and it succeeds when all medicinal agents have failed."

We can have no doubt that this method of dealing with pulmonary tuberculosis will be largely tried in England. An elevated position so as to be beyond the range of evening fogs will, of course, be an advantage, but it would seem that under almost all conditions the outer air is better than the air of the house. The chief difficulty in arranging for an out-door treatment is likely to arise from the fact that if it is to be of its full utility it cannot well be combined with the ordinary indoor life of the rest of the family. This is no trouble at an institution where all is arranged for the invalid for the purpose of the "cure," but in attempting to carry out the treatment at home it will certainly add greatly to the complexity of the problem. The invalid who has been passing his day in the frosty air, and is about to pass his night with his windows wide open, certainly must not spend his evening in the bosom of his family amid the cosy comforts of the drawing-room fire-side. Much consideration and much self-sacrifice will of necessity be entailed on the relatives in providing society and recreation for the invalid. All this, however, will be avoided in institutions, and it seems not unlikely that the problem of isolating cases of tuberculosis, which has hitherto seemed to be surrounded by many difficulties, will in the end solve itself by the fact becoming generally recognised that it is not a question concerning the friends alone, but that even for the patients it is best for those who suffer from consumption to live in special institutions.

